# Translation of c-Met Targeted Image-Guided Surgery Solutions in Oral Cavity Cancer—Initial Proof of Concept Data

**DOI:** 10.3390/cancers13112674

**Published:** 2021-05-28

**Authors:** Tessa Buckle, Maarten van Alphen, Matthias N. van Oosterom, Florian van Beurden, Nina Heimburger, Jaqueline E. van der Wal, Michiel van den Brekel, Fijs W. B. van Leeuwen, Baris Karakullukcu

**Affiliations:** 1 Interventional Molecular Imaging Laboratory, Department of Radiology, Leiden University Medical Center, 2333 ZA Leiden, The Netherlands; m.n.van_oosterom@lumc.nl (M.N.v.O.); f.van_beurden@lumc.nl (F.v.B.); n.heimburger@lumc.nl (N.H.); f.w.b.van_leeuwen@lumc.nl (F.W.B.v.L.); 2 Department of Head and Neck Surgery and Oncology, Netherlands Cancer Institute-Antoni van Leeuwenhoek Hospital, 1066 CX Amsterdam, The Netherlands; m.v.alphen@nki.nl (M.v.A.); m.v.brekel@nki.nl (M.v.d.B.); b.karakullukcu@nki.nl (B.K.); 3 Department of Pathology, Netherlands Cancer Institute-Antoni van Leeuwenhoek Hospital, 1066 CX Amsterdam, The Netherlands; j.vd.wal@nki.nl

**Keywords:** molecular imaging, fluorescence, image-guided surgery, c-Met, head and neck cancer

## Abstract

**Simple Summary:**

Translation of tumor-specific fluorescent tracers is crucial in the realization intraoperative of tumor identification during fluorescence-guided surgery. Ex vivo assessment of surgical specimens after topical tracer application has the potential to reveal the suitability of a potential surgical target prior to in vivo use in patients. In this study, the c-Met receptor was identified as a possible candidate for fluorescence-guided surgery in oral cavity cancer. Freshly excised tumor specimens obtained from ten patients with squamous cell carcinoma of the tongue were incubated with EMI-137 and imaged with a clinical-grade Cy5 prototype fluorescence camera. In total, 9/10 tumors were fluorescently illuminated, while non-visualization could be linked to non-superficial tumor localization. Immunohistochemistry revealed c-Met expression in all ten specimens. Tumor assessment was improved via video representation of the tumor-to-background ratio.

**Abstract:**

Intraoperative tumor identification (extension/margins/metastases) via receptor-specific targeting is one of the ultimate promises of fluorescence-guided surgery. The translation of fluorescent tracers that enable tumor visualization forms a critical component in the realization of this approach. Ex vivo assessment of surgical specimens after topical tracer application could help provide an intermediate step between preclinical evaluation and first-in-human trials. Here, the suitability of the c-Met receptor as a potential surgical target in oral cavity cancer was explored via topical ex vivo application of the fluorescent tracer EMI-137. Freshly excised tumor specimens obtained from ten patients with squamous cell carcinoma of the tongue were incubated with EMI-137 and imaged with a clinical-grade Cy5 prototype fluorescence camera. In-house developed image processing software allowed video-rate assessment of the tumor-to-background ratio (TBR). Fluorescence imaging results were related to standard pathological evaluation and c-MET immunohistochemistry. After incubation with EMI-137, 9/10 tumors were fluorescently illuminated. Immunohistochemistry revealed c-Met expression in all ten specimens. Non-visualization could be linked to a more deeply situated lesion. Tumor assessment was improved via video representation of the TBR (median TBR: 2.5 (range 1.8–3.1)). Ex vivo evaluation of tumor specimens suggests that c-Met is a possible candidate for fluorescence-guided surgery in oral cavity cancer.

## 1. Introduction

Fluorescence-guided applications in head-and-neck surgery are at the forefront of the progression realized in this field [1,2,3,4]. The most common fluorescence guidance applications in head and neck surgery are implemented during the surgical resection of sentinel nodes, a secondary means of identifying metastatic tumor spread [1,2,5,6]. According to EANM guidelines for sentinel node localization in oral cavity cancer [7], imaging of lymphatic drainage pathways can provide both a preoperative roadmap as well as intraoperative guidance towards micro metastatic lesions in the part of the lymphatic system that is associated with the primary tumor. Technical (r)evolutions in (radio)pharmaceutical design and surgical imaging modalities have advanced sentinel node resection and have provided insight into the tracer doses required for surgical guidance [1,2]. The holy grail in image-guided surgery, however, is direct identification of the primary tumor (margins) and/or (macro)metastases. This is based on the assumption that, through the direct illumination of tumor cells, image guidance supports intraoperative tumor delineation [8] and could aid in the resection of lesions that could not be identified otherwise [9].

Although broadly explored in the preclinical setting [10,11], the application of tumor-specific tracers in clinical trials is still in its infancy. While clinical trials are performed using an increasing array of targets and matching fluorescence tracers, the epidermal growth factor receptor (EGFR) appears to be most widely studied [6,12,13,14]. Given the complexity of receptor over-expression and heterogeneity among head and neck cancers (e.g., melanoma and oral squamous cell carcinoma (OSCC)), one may wonder if the available number of tracer candidates should not be extended. Expansion of the arsenal of tracers seems to be limited by a translational gap between preclinical work in artificial tumor models and in vivo implementation in clinical trials. We, and others, have argued that receptor-targeting tracers can be evaluated using an ex vivo topical application setting first, using fresh surgical samples [8,15]. Such evaluations help circumvent the notoriously poor correlation between mRNA and protein expression and cytoplasmic target presence using immunohistochemistry (IHC; [16,17,18]). This approach, however, cannot solve the intricate pharmacokinetic challenges of tracer design that generally tend to focus on GMP-compatible synthesis, targeting affinity and biodistribution [19].

The role of the mesenchymal-epithelial transition factor (c-Met) in the progression of head and neck cancer is well described [20,21]. C-Met plays an important role in tumor development [20]. Furthermore, its over-expression is stated to be linked to poor pathological features and prognosis [21]. Immunohistochemical assessment of c-Met in squamous cell carcinoma (SCC), the most frequent form of cancer in the head and neck area, shows expression in 78%–82.9% of tumors [21,22,23]. In OSCC, c-Met overexpression is substantially higher than in normal mucosa located adjacent to the tumor [20]. Together with the multiple examples wherein c-Met is being exploited for therapeutic purposes [24], this implies that c-Met could also serve as an imaging target for OSCC. EMI-137 is a c-Met targeting fluorescent tracer that, following extensive tracer optimization and preclinical evaluations, has been translated to the clinic and comes with thoroughly documented pharmacokinetics [9]. This tracer also previously demonstrated its ability to apply c-Met targeted resection of colorectal polyps, Barret’s neoplasia and penile cancer, based on far-red related fluorescence after intravenous administration [8,9,25].

**Figure 1:**
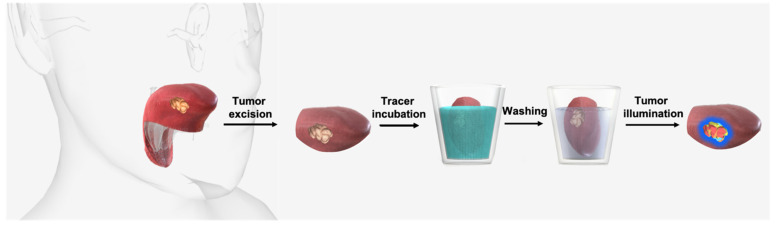
Tumor incubation and illumination. Schematic overview of the workflow from tumor excision to illumination. After excision, the tongue tumor sample was incubated in a solution containing EMI-137. After washing to eliminate unbound tracer, fluorescence imaging allowed visualization of the tumor within the specimen.

**Figure 2:**
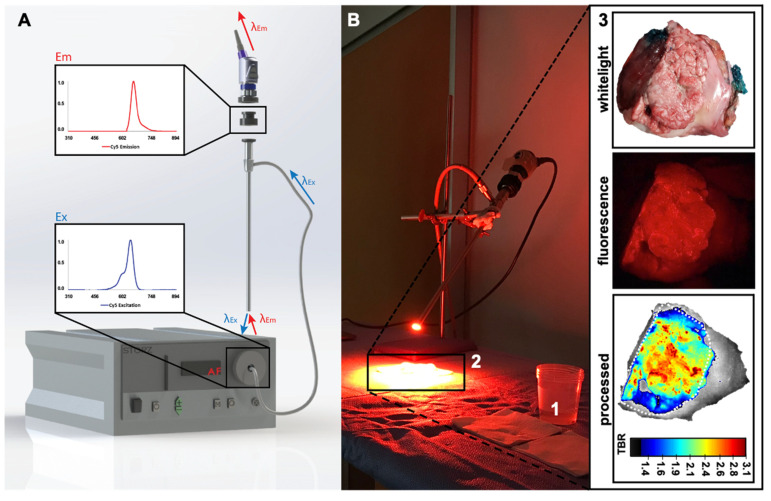
Imaging set-up and image processing. (**A**) The dedicated Cy5 imaging system consisting out of a Karl Storz light-source (I), fluorescence camera (II), Cy5 clip-on filter (III) and scope (IV). Inserts show the fluorescence excitation (bottom; λ_ex_ 650 nm) and emission (top; λ_em_ 670 nm) of the far-red dye Cy5. (**B**) Tracer incubation (**1**) and camera set-up for imaging of the tissue sample (**2**) of a representative tissue sample (**3**) showing the location of the tumor (dotted line) using white light (**top**) and standard fluorescence imaging (**center**) and after image processing (**bottom**; the scale bar represents the tumor-to-background ratio (TBR) corresponding to the fluorescence signal intensity).

To address the need for rapid translation of tumor-targeted image-guided surgery solutions for oral cavity cancer, we explored c-Met as a potential surgical target in OSCC. These initial evaluations were based on ex vivo incubation of fresh surgical tongue tumor specimens with EMI-137 (Figure 1), which was subsequently imaged with a clinical-grade Cy5 prototype fluorescence camera system (Figure 2).

## 2. Materials and Methods

### 2.1. Patients

Ten patients with OSCC of the tongue were prospectively included for ex vivo evaluation of the feasibility of c-Met-targeted tumor identification using fluorescence imaging. Primary tumor type, clinical TNM stage, tumor location, pathological TNM stage and differentiation grade were recorded. Approval for the study was obtained from the institutional review board, and all patients signed an informed consent that approved the use of excised tissue specimens for research purposes.

### 2.2. Tracer Preparation

For ex vivo sample assessment, the c-Met targeting Cy5 fluorescent tracer EMI-137 was dissolved in PBS to a concentration of 25, 250, 500 or 2500 nmol (20 mL per sample).

### 2.3. Ex Vivo Incubation of Tissue and Fluorescence Imaging

Resected tumor specimens were collected directly post-excision and bisected, and the fresh (non-fixed) samples were incubated with EMI-137 (Figure 1). Hereafter, samples were rinsed twice with PBS to clear unbound EMI-137, and the specimen was imaged directly after. The first three specimens were incubated in an increasing concentration of EMI-137. Rinsing and imaging were performed after each incubation step. The optimization of the imaging time was assessed by imaging at different intervals (1, 2 and 5 min) during incubation. The assessment of the most suitable concentration for incubation was performed based on fluorescence intensity and uptake in healthy tissue. The remaining samples were evaluated using the optimized protocol.

### 2.4. Fluorescence Imaging

White light and far-red fluorescence imaging were conducted using a clinical-grade Cy5 prototype (Karl Storz Endoskope GmbH, Tuttlingen, Germany [26]; Figure 2A). This set-up was complemented with in-house developed image-processing software that allowed color-coding to be directly related to the tumor-to-background ratio (TBR; ratio between relative fluorescence units [27] in the tumor and surrounding tissue) via the creation of a rainbow colormap or heatmap. This heatmap allowed direct real-time visualization of the distribution of the fluorescence signal (pseudo-colored fluorescence overlay) within the tissue sample (visible on a separate screen) and representation of the TBR via an intensity-based scalebar (fluorescence signal intensity differences represented via a color spectrum; Figure 2(B3)). Image-processing software was written in C++-programming language using open-source computer vision libraries (OpenCV). Confirmation of the TBR values was obtained through manual assessment of the fluorescence images using ImageJ software (based on measurement of intensity in 10 different regions of interest throughout the sample, including tumor and surrounding tissue).

### 2.5. Pathological Assessment

After ex vivo imaging, the specimen was formalin-fixed, sectioned and then paraffin-embedded. IHC of the formalin-fixed paraffin-embedded tumor samples was performed on a BenchMark Ultra autostainer (Ventana Medical Systems, Oro Valley, AZ, USA). In brief, 3 µm paraffin sections were deparaffinized with the EZ prep solution (Ventana Medical Systems), and heat-induced antigen retrieval was carried out using Cell Conditioning 1 (Ventana Medical Systems). Hereafter, sections were incubated with an anti-c-MET antibody (clone SP44; Roche Diagnostics, Rotkreuz, Switserland), and an UltraView Universal DAB Detection Kit (Ventana Medical Systems) was used to visualize the c-Met expression. Slides were counterstained with Hematoxylin and Bluing Reagent (Ventana Medical Systems). Due to the lack of a quantitively read-out in routine immunohistochemistry, the c-Met expression levels were visually scored by a dedicated pathologist. Scoring was based on the intensity of the UltraView signal (four classifications were used: no staining, weak, moderate and strong, respectively −, +, ++, +++). Both membrane and cytoplasmic staining were noted. Since c-Met is considered a membrane-bound biomarker [9], membranous staining was leading in the scoring.

## 3. Results

### 3.1. Patients

Patient and tumor characteristics are presented in Table 1. Tumors were all located in the lateral tongue. Nine patients underwent removal of the primary tumor, and one patient underwent a re-excision. Clinical TNM stage ranged from T1 to T3 (T1: *N* = 5, T2: *N* = 4 and T3: *N* = 1).

#### Ex Vivo Incubation and c-Met Related Fluorescence Imaging

During ex vivo assessment, the focus was placed on the primary tumor (Figure 1). Incubation of tissue specimens with different quantities of EMI-137 (25–2500 nmol) indicated that the best TBR was obtained using concentrations ≥ 250 nmol. Since incubation with 500 nmol yielded the best TBR’s, 500 nmol was selected as the optimal concentration for incubation. Imaging at different time points during the incubation revealed that incubation time also had an impact. As the TBR’s were highest after 5 min of incubation, this time point was used during further investigations.

**Figure 3:**
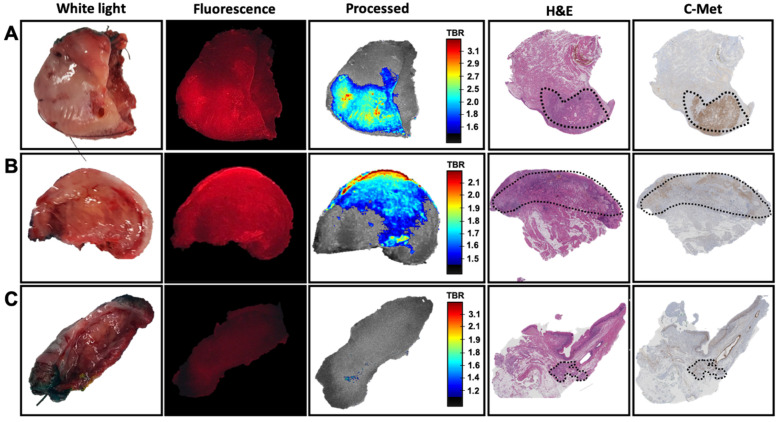
The correlation between c-Met targeted fluorescence and immunohistochemistry. Overview of imaging and pathological assessment of three different samples showing ex vivo white light, Cy5-based fluorescence imaging of the incubated tissue (fluorescence in red), real-time image processing of the fluorescence signal (including video representation of tumor-to-background ratio (TBR)), H&E staining and c-MET immunohistochemistry. (**A**) superficial tumor localization, (**B**) tumor presence on the cleavage plane and (**C**) a sample without tumor presence on the cleavage plane. Delineation of the tumor (dashed) was based on H&E.

After incubation with EMI-137 (Figure 1 and Figure 2(B1)), a tumor-related fluorescence signal could be detected in 9/10 samples (Table 1, Figure 2(B3) and Figure 3A,B). Visual assessment of the one sample wherein the tumor could not be identified using fluorescence imaging revealed that there was no tumor present on the incubated cleavage plane (Figure 3C, white light image). Pathological assessment of this sample revealed tumor presence in less superficial areas in tissue sections that did not correspond with the incubated cleavage plane (Figure 3C, H&E and c-Met staining).

Without processing, the Cy5-related fluorescence was presented as red-on-black (Figure 2B and Figure 3). This allowed visualization of the uptake of EMI-137 in the sample. Real-time image processing using intensity-based rainbow color mapping enhanced the utility of these fluorescence images. This approach provided improved visual discrimination between tumor and healthy tissue, but the color-coded visualization also yields an intuitive rate interpretation of the TBR values (ranging from blue for low TBR to red for the highest TBR within the sample). The median TBR was 2.5 (range 1.8–3.1; Table 1), underlining the effective discrimination of the tumor from the surrounding tissue. The same visualization technique also provided improved insight into the heterogeneity within the lesion (Figure 2B and Figure 3). Overall, expansion of fluorescence outside the primary tumor did not exceed TBR > 2.

The pathological assessment provided a TNM stage ranging between T1 and T2 (T1: *N* = 6, T2: *N* = 4; Table 1). The presence of EMI-137 in the tissue sample had no adverse effect on the further pathological assessment of the tissue. IHC revealed the expression of c-Met in 10/10 samples (Table 1) with a c-Met status ranging between + and +++. The highest number of samples showed expression levels of 1+ (3/10) or 1+/2+ (4/10). A clear overlap could be observed between the accumulation of EMI-137 and the c-Met expression levels identified using IHC (Figure 3). Furthermore, intensity differences seen in the fluorescence images could be correlated to heterogeneity in c-Met expression throughout the lesion and/or diffuse tumor presence throughout the tissue specimen. Here, it must be noted that some distortion between the fluorescence imaging on fresh tissue and the immunohistochemical analysis of fixates coupes was seen, which was caused by the deformation of the tissue during fixation. Unfortunately, the loss of a fluorescence signal in the embedded tissue samples (due to sample pre-treatment) meant that for fluorescence imaging, we needed to rely on the data obtained using the fresh tissue specimens. As a result, it becomes impossible to provide a direct overlay between fresh and fixed tissue samples. This limited our ability to finetune the scaling of the TBR visualization.

## 4. Discussion

Ex vivo assessment of fresh tissue samples obtained after oral cavity surgery provided the next step towards the realization of in vivo c-Met guided tumor surgery in the head-and-neck area. This set-up successfully allowed the assessment of using c-Met as an imaging target in OSCC.

C-Met targeted fluorescence imaging, in combination with image processing, was shown to enable signal intensity-based identification in 90% of the primary tumor samples based on the representation of the TBR (Figure 2B and Figure 3). However, c-Met expression was observed in all tumor specimens at IHC (Table 1). This mismatch seems to be caused by the non-superficial tumor localization on either the surface of the tongue or the cleavage plane (Figure 3C). Both fluorescence imaging and ex vivo tissue incubation can be impaired by tissue overlying the tumor. In the case of fluorescence imaging, it is well documented that tissue attenuates and scatters the fluorescence signal [26]. While for tissue incubation the maximum depth of tracer penetration is not yet well understood, there are studies that show that at least several cell layers (up to 300 µm) can be effectively stained and evaluated [28,29]. And while physics will dictate the superficial nature of fluorescence imaging, in vivo use of EMI-137 suggest that the issues behind tracer penetration in tissue are overcome when applying EMI-137 (or any other fluorescent tracer) in humans [8,9,25]. More specifically, in penile cancer patients, the presence of fluorescence was not limited to the rim of the tumor but could be detected throughout the tumor (tumor infiltration depth up to 24 mm [8]). This utility of EMI-137 is strengthened by other critical findings. For example, in colorectal cancer patients, EMI-137 allowed for the identification of lesions that were missed using white-light endoscopy [9]. Additionally important, the clinical implementation of EMI-137 fluorescence guidance did not interfere with the standard clinical surgical and pathological workflow of Barret’s neoplasia and penile cancer [8,25].

While the fluorescence signal is often presented using a pseudo-color (overlay) image [27], the standard procedure is to calculate the TBR at some point after the acquisition of the images. Real-time image processing not only provided increased discrimination between the tumor and surrounding tissue (Figure 2 and Figure 3), but it also allowed assessment of fluorescence images to go from qualitative pseudo-colored fluorescence overlay images towards more quantitative analysis of the TBR in real-time. This improvement in signal representation also enabled assessment of the distribution of the fluorescence signal throughout the tissue sample, and thus the heterogeneity in c-Met expression. As real-time fluorescence imaging is said to enable improved decision making during tumor resection [27], real-time signal quantification (and representation) has the potential to further revolutionize its applicability. Obviously, scaling of the fluorescence images impacts image perception, and a clear cut-off point in the signal would help enables discrimination between the tumor and the surrounding tissue. Where some state that TBR values higher than the theoretical value of two are only of value, the human eye is sensitive enough to identify lower TBR values [27]. As such, setting a clinically relevant cut-off point for the fluorescent signal intensity is challenging to say the least.

According to the IUPAC regulations, the fluorescent emission used for surgery is classified into: visible fluorescence (400 nm–650 nm), far-red fluorescence (650 nm–780 nm) and NIR fluorescence (780–2000 nm [30]). While NIR-based fluorescence imaging is popular amongst many that pursue the development of new tracers for fluorescence-guided surgery, far-red Cy5-based tracers are also on the rise in clinical trials, e.g., based on EMI-137 [8,9,25] and cRGDY-PEG-Cy5.5-nanoparticles [6]. In fact, visible dyes, such as fluorescein and pPXi, have been widely applied in clinical trials [31]. Beyond that, literature even suggests that fluorescent emissions in different portions of the fluorescent spectrum can complement each other in multi-wavelength fluorescence imaging [26,31].

In the small sample size included in this study, c-Met expression was seen in all of the tumor samples, but lower percentages (70%–78%) are reported in various tumor locations in the head and neck area [32,33,34]. An explanation for this discrepancy can be found in (1) differences in expression between tumors of different locations and variations that occur in c-Met expression during tumor progression and (2) variation in the standards used for interpretation of the immunohistochemical results due to the lack of a universally accepted scoring principle. With regard to the first, c-Met expression has been assessed in various tumor types in the head and neck (82.9% in OSCC (exact location not specified; [33]), 70% in hypopharyngeal cancer [32] and 54.9% in laryngeal carcinoma [35]). OSCC of the tongue (Table 1) has not been specifically assessed previously and could thus vary, similar to the variation seen in c-Met expression in different types of lung cancer (range 67%–100% [36]). With regard to the second point, varying standards are used to determine the level of c-Met expression via immunohistochemistry, ranging from > 10%, 30% or 50% of cells to be stained before being deemed c-Met positive [22,32,37], while the use of a scoring system based on intensity categories as was used in this study is also not uncommon [32]. In addition, it is worth noting that cytoplasmic staining is often also included in the assessment of staining, while only membranous staining is in agreement with requirements for receptor-targeted imaging purposes [28].

In this study, the focus was placed on c-Met as an imaging target, whereas we are confident that the ex vivo set-up described can be applied in a more generic fashion, meaning for a wide range of targets, tumor types and fluorescent emissions. Besides revealing the potential of ex vivo tissue evaluation as an intermediate step in the translational process, this study clearly also has its limitations. One is the small sample size of this proof-of-concept study. The obvious next step is to corroborate these results in a larger group and in vivo. In penile cancer patients, the feasibility of in vivo targeting based on ex vivo tissue evaluation in a limited number of samples (*n* = 10) has been shown to allow successful translation of receptor-targeted imaging concepts [8]. Further investigations will also be focused on the possibility of setting TBR cut-off values and the identification of microscopic lesions via a more detailed assessment of the fluorescence uptake. Lastly, targeted approaches, such as c-Met targeting, may benefit from patient selection based on patient-specific characteristics. To this end, evaluation of the hypothesis that especially the patients with a worse tumor stage or human papillomavirus induced tumors might benefit from c-MET-targeted surgery [8] might be warranted, though further evaluation of the relation between the c-Met score, TBR and the T-stage and metastatic potential.

## 5. Conclusions

Ex vivo evaluation of tongue tumor specimens suggests that c-Met is a potential candidate target for fluorescence-guided surgery in oral squamous cell cancer/oral cavity cancer.

## Figures and Tables

**Table 1:** Patient characteristics.

Patient No	Sex	Age	Tumor Type	Clinical T-Stage	PA T-Stage	Fluorescence in Tumor (Y/N)	TBR	c-Met Status *
1	M	72	OSCC	T1	T1	Y	1.8 ± 0.3	+
2	M	62	OSCC	T3	T2	Y	2.5 ± 0.7	+/++
3	M	56	OSCC	T2	T2	Y	2.0 ± 0.1	+/++
4	F	86	OSCC	T1	T1	Y	2.5 ± 0.5	+
5	F	80	OSCC	T2	T1	Y	2.4 ± 0.3	++/+++
6	F	67	OSCC	T2	T2	Y	2.7 ± 0.3	+
7	M	68	OSCC	T1	T1	Y	3.1 ± 0.7	+/++
8	F	68	OSCC	T2	T2	Y	2.3 ± 0.8	+++
9	F	76	OSCC	T1	T1	N	N.A.	++
10	M	36	OSCC	T1	T1	Y	2.5 ± 0.5	+/++

OSCC: oral squamous cell carcinoma. TBR: Tumor-to-background ratio. N.A.: Not available. No tumor detected; therefore, ratio fluorescence signal in tumor and surrounding healthy tissue could not be evaluated. * C-Met status as identified on immunohistochemistry.

## Data Availability

Data can be made available upon reasonable request.
